# Optimal fractal tree-like microchannel networks with slip for laminar-flow-modified Murray’s law

**DOI:** 10.3762/bjnano.9.46

**Published:** 2018-02-08

**Authors:** Dalei Jing, Shiyu Song, Yunlu Pan, Xiaoming Wang

**Affiliations:** 1 School of Mechanical Engineering, University of Shanghai for Science and Technology, Shanghai, 200093, P. R. China; 2 Key Laboratory of Micro-Systems and Micro-Structures Manufacturing, Ministry of Education and School of Mechatronics Engineering Harbin Institute of Technology, Harbin, 150001, P. R. China,; 3 School of Electrical Engineering and Automation, Harbin Institute of Technology, Harbin, 150001, P. R. China

**Keywords:** fractal tree-like microchannel networks, hydraulic resistance, Murray’s law, slip

## Abstract

The fractal tree-like branched network is an effective channel design structure to reduce the hydraulic resistance as compared with the conventional parallel channel network. In order for a laminar flow to achieve minimum hydraulic resistance, it is believed that the optimal fractal tree-like channel network obeys the well-accepted Murray’s law of β_m_ = *N*^−1/3^ (β_m_ is the optimal diameter ratio between the daughter channel and the parent channel and *N* is the branching number at every level), which is obtained under the assumption of no-slip conditions at the channel wall–liquid interface. However, at the microscale, the no-slip condition is not always reasonable; the slip condition should indeed be considered at some solid–liquid interfaces for the optimal design of the fractal tree-like channel network. The present work reinvestigates Murray’s law for laminar flow in a fractal tree-like microchannel network considering slip condition. It is found that the slip increases the complexity of the optimal design of the fractal tree-like microchannel network to achieve the minimum hydraulic resistance. The optimal diameter ratio to achieve minimum hydraulic resistance is not only dependent on the branching number, as stated by Murray’s law, but also dependent on the slip length, the level number, the length ratio between the daughter channel and the parent channel, and the diameter of the channel. The optimal diameter ratio decreases with the increasing slip length, the increasing level number and the increasing length ratio between the daughter channel and the parent channel, and decreases with decreasing channel diameter. These complicated relations were found to become relaxed and simplified to Murray’s law when the ratio between the slip length and the diameter of the channel is small enough.

## Introduction

For the microfluidic systems widely used in various fields such as lab-on-a-chip for chemical and biomedical detection and microchannel heat sinks for the cooling of electronic systems [1–2], the optimal mass and heat transfer performance is essential, yet challenging. For example, a microchannel heat sink has a better convective heat transfer performance, but needs a larger pumping power. Thus, the optimal design of the channel layout to achieve better mass and heat transfer performance is needed. Fractal tree-like branched networks are found abundant in nature, for example, in the vascular systems of animals, in tree branches and leaf veins of plants, all of which can provide inspiration for the optimal design of the channel layout to achieve the optimal mass and heat transfer [3–5]. These branched networks are known to have excellent performance in transport processes of heat and mass, and have been widely studied and used in the fields of fluid flow, heat conduction, and heat convection [6–18].

For fluid flow, it is found that the fractal tree-like channel networks require less pumping power and have a smaller hydraulic resistance as compared with the conventional parallel channel systems under the constraint of the same channel volume. Furthermore, the optimal structure of the fractal tree-like channel networks for fluid flow to achieve minimum hydraulic resistance obeys the well-accepted Murray’s law in the manner of β_m_ = *N*^Δ^, where β_m_ is the diameter ratio between the daughter channel and the parent channel, *N* is the branching number, Δ is a parameter relating to the different practical applications. It has been found that Δ = –1/3 for laminar flow and −7/3 for turbulent flow [12,14–18].

**Figure 1:**
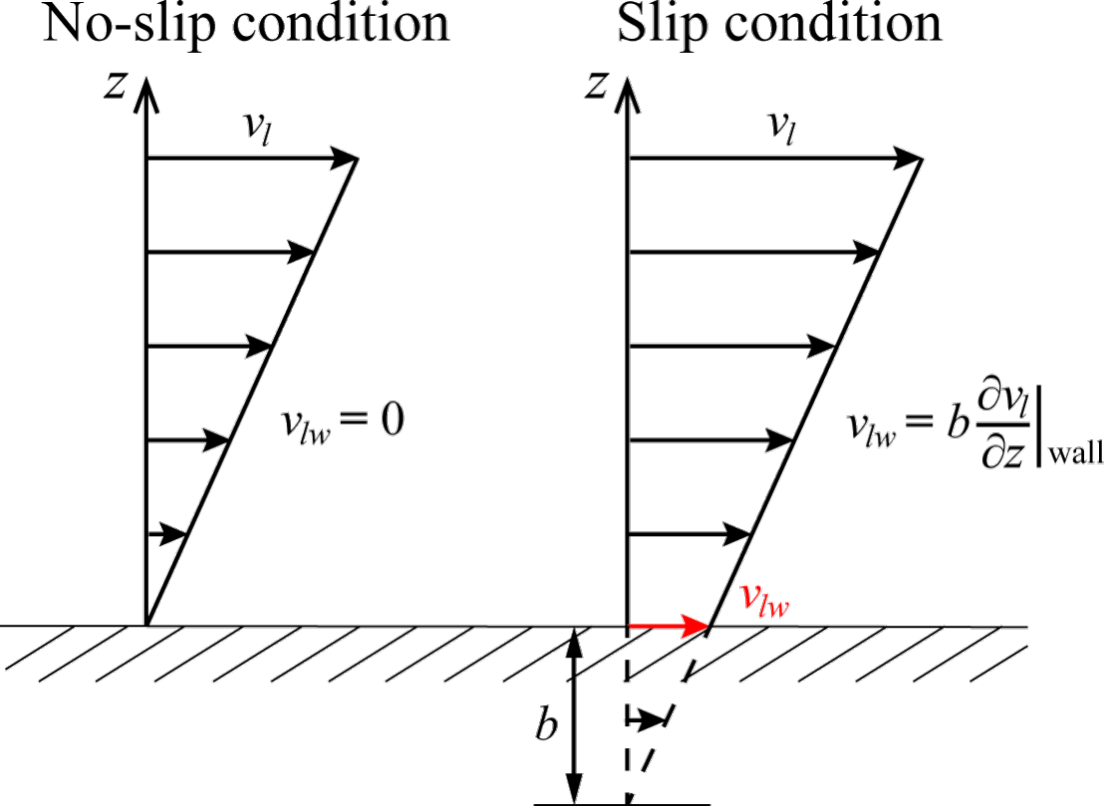
Simplified schematic of the no-slip and slip conditions. ν_lw_ is the relative velocity between the wall and the adjacent liquid, ν_l_ is the velocity field of the liquid, *b* is the slip length, and 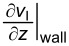 is the liquid velocity gradient in the direction perpendicular to the wall.

Although Murray’s law has been verified by numerous theoretical and experimental studies, it is obtained under a no-slip condition assumption at the channel wall–liquid interface [12,14–18]. This no-slip condition assumption is reasonable for fluid flow in a macroscale channel, however, for a microscale channel, this assumption is debatable, because it has been found that there is another hydrodynamic condition, the slip condition, at some solid–liquid interfaces [19–23]. Figure 1 gives a simplified schematic of the no-slip condition and the slip condition, indicating that a significant difference between the no-slip condition and the slip condition is the relative velocity between the solid wall and the adjacent liquid. For the slip condition, the relative velocity is non-zero and the degree of slip is manifested by the slip length *b* at the solid wall–liquid interface as follows [20–23]

[1]
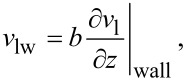


where ν_lw_ is the relative velocity between the solid wall and the adjacent liquid, and 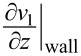 is the liquid velocity gradient in the direction perpendicular to the solid wall. The reported value of the slip length at various solid–liquid interfaces varies from tens of micrometers down to several nanometers [19,21–23]. Thus, the effect of slip on the macroscale fluid flow is usually weak and negligible; however, for microscale fluid flow, slip is believed to effectively reduce the hydraulic resistance of fluid flow and should be considered [23–26]. Nevertheless, there is less study on the fluid flow in the fractal tree-like microchannel network considering the slip condition.

To solve this problem, the present work reinvestigates the laminar flow in a fractal tree-like microchannel network considering the slip condition. The optimal structure of a fractal tree-like microchannel network with slip for laminar flow to achieve minimum hydraulic resistance is analyzed. The effects of slip length, the structural and dimensional parameters (including the branching number, the level number, the length and the diameter of channel) on the optimal fractal tree-like microchannel networks with slip condition are also studied.

## Modeling

### Generation of fractal tree-like microchannel networks

**Figure 2:**
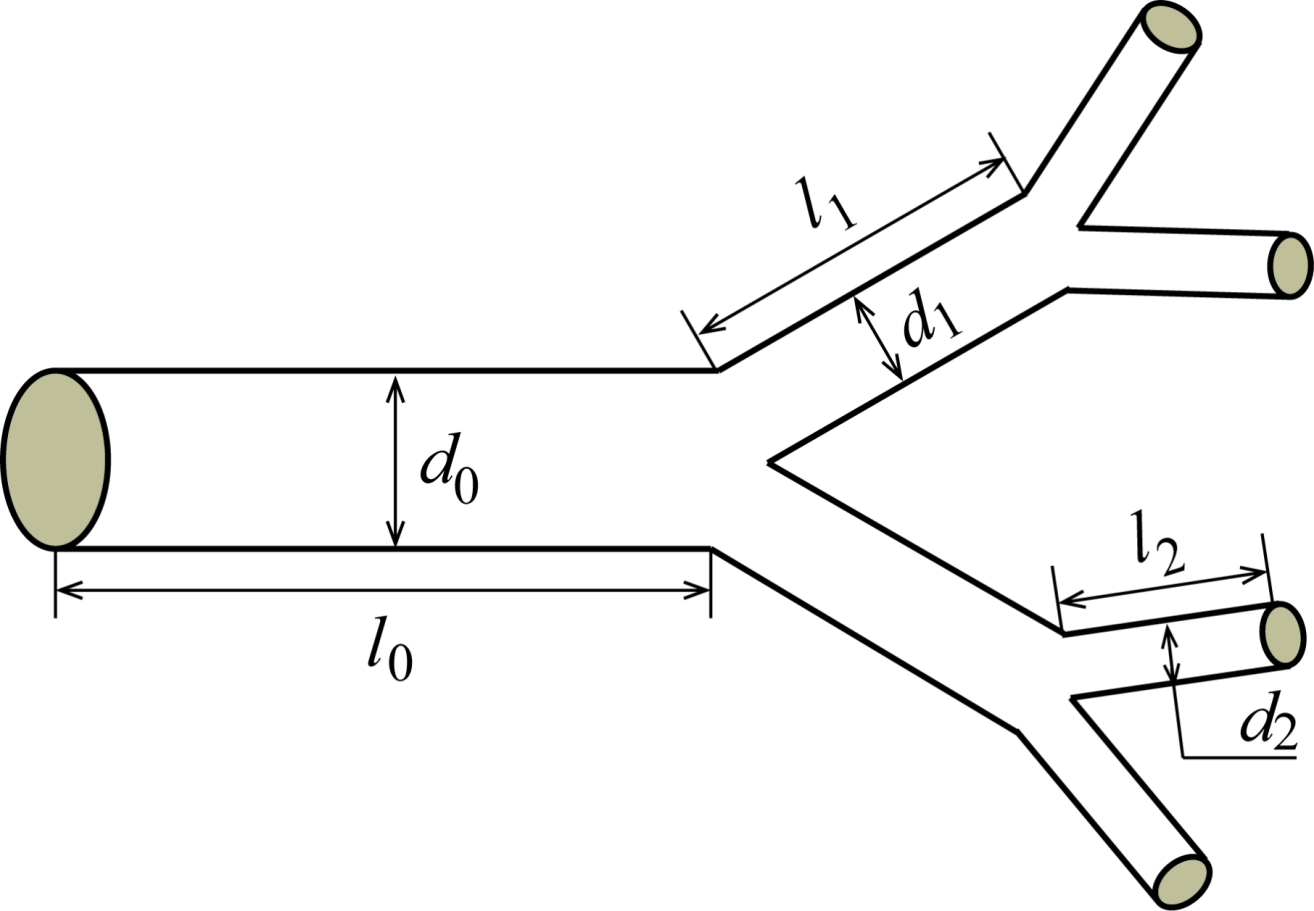
Schematic of a self-similar fractal tree-like microchannel network with branching number *N* = 2 and total number of branching levels *m* = 2. *l_k_* (*k* = 0, 1, 2) is the length of the microchannel at the *k*th level and *d_k_* (*k* = 0, 1, 2) is the hydraulic diameter of the microchannel at the *k*th level.

For different applications of a fractal tree-like branched network, a typical structure similar to that shown in Figure 2 has been widely used [6–18]. In the present work, a similar structure of the fractal tree-like microchannel network is used to study the transportation of fluid flow. This similar fractal tree-like microchannel network is generated as follows. (1) From the 0th branching level with a single microchannel, every microchannel is divided into *N* branches with the same length and hydraulic diameter at the following level (e.g., *N* = 2 for the case in Figure 2). (2) The geometric dimensions of every single microchannel at a certain level satisfy the following scaling law,

[2]
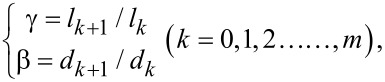


where γ is the length ratio between the microchannel at the (*k*+1)th level and the microchannel at the *k*th level, *l_k_* is the length of the microchannel at the *k*th level, β is the hydraulic diameter ratio between the microchannel at the (*k*+1)th level and the microchannel at the *k*th level, *d_k_* is the hydraulic diameter of the microchannel at the *k*th level, and *m* is the total number of branching levels.

From Equation 2, the following equations can be easily obtained,

[3]
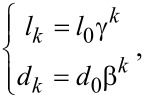


where *l*_0_ and *d*_0_ are the length and hydraulic diameter of the microchannel at the 0th level, respectively. During the generation of the fractal tree-like microchannel network, the values of *l*_0_ and *d*_0_ are usually known. The fractal tree-like branched network generated in this manner is a symmetric and self-similar network.

### Theoretical model

In this section, the pressure-driven flow in the fractal tree-like microchannel network generated in the last section will be modeled. Before the modeling, the following assumptions are made. (1) Every single microchannel is a smooth, cylindrical tube and the thickness of the channel wall is thin enough to be neglected. (2) The ratio between the length and diameter of every single microchannel is large enough to make sure the fluid flow is in steady state. (3) The fluid flow in every single microchannel is incompressible, laminar, fully developed Newtonian flow. (4) The effect of junctions on the pressure drop is neglected. (5) The wall of every single channel suffers the same slip length.

Based on the above assumptions together with the Hagen–Poiseuille equation, the hydraulic resistance of fluid flow in any single microchannel with boundary slip at the *k*th level can be expressed as follows [26]

[4]
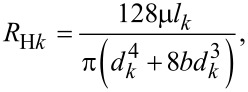


where *R*_H_*_k_* is the hydraulic resistance of fluid flow in any single microchannel at the *k*th level, and μ is the dynamic viscosity of the fluid flow.

For the pressure-driven flow in a fractal tree-like microchannel network with *N* branches and *m* levels, the total hydraulic resistance of the fluid flow in the entire network can be obtained using the electric circuit analogy based on the physical similarities between microfluidics and electronics as follows [17,27].

[5]
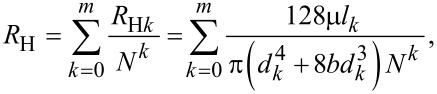


where *R*_H_ is the total hydraulic resistance of the fluid flow in the entire network.

Introducing Equation 3 into Equation 5, the total hydraulic resistance can be further expressed as

[6]
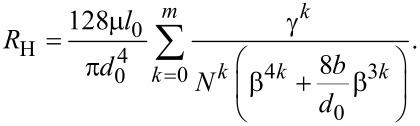


In order to carry out the optimal analysis of the fractal tree-like microchannel network with slip to achieve the minimum hydraulic resistance, an equivalent single microchannel is introduced as a reference based on a similar method to that presented in the literature [11–12,17]. Furthermore, the equivalent single microchannel is assumed to have the same volume and length as the entire network. This is because in the self-evolution of natural fractal tree-like systems (or the optimal design of a manufactured fractal tree-like channel network for fluid flow to achieve minimum hydraulic resistance) the total volume and length of the network are usually kept constant as constraints. In addition, the equivalent single microchannel is assumed to suffer under no-slip condition when analyzing the effect of slip on the optimal design of the tree-like network and its hydraulic resistance. Thus, the hydraulic resistance of the equivalent single microchannel, *R*_He_, can be expressed as follows.

[7]
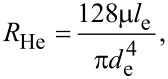


where *l*_e_ is the equivalent length of the equivalent single microchannel, and *d*_e_ is the equivalent diameter of the equivalent single microchannel. Based on the assumption that the equivalent single microchannel has the same length as the entire network, the equivalent length of the equivalent single microchannel is given as [11–12,17]

[8]
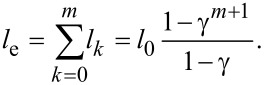


Because the equivalent single microchannel has the same volume as the entire network, the equivalent diameter of the equivalent single microchannel can be obtained as follows [11–12,17]

[9]
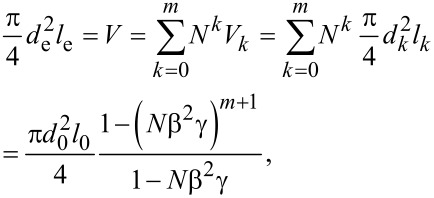


where *V* is the total volume of the entire network and *V_k_* is the volume of every single microchannel at the *k*th level of the network. Thus, the equivalent diameter of the equivalent single microchannel can be expressed as,

[10]
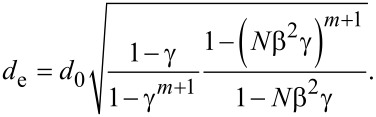


Combining Equations 6–10, the dimensionless hydraulic resistance of the laminar flow in the fractal tree-like microchannel network can be expressed as,

[11]
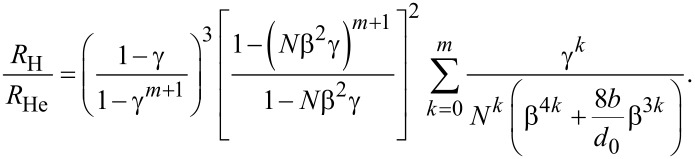


From Equation 11, the dimensionless hydraulic resistance of the laminar flow in the fractal tree-like microchannel network is dependent on the slip length, the structural and the dimensional parameters (γ, β, *N*, *m*, *d*_0_). Equation 11 can be used to analyze the effects of different parameters on the hydraulic resistance of fluid flow in a tree-like microchannel network and to optimize the structure of the fractal tree-like microchannel network for fluid flow to achieve the minimum hydraulic resistance. Actually, Equation 11 can be considered as a comprehensive model to analyze the hydraulic resistance of laminar flow in the fractal tree-like microchannel network with both no-slip (*b* = 0) and slip conditions (*b* ≠ 0) and to compare their difference.

## Results and Discussion

**Figure 3:**
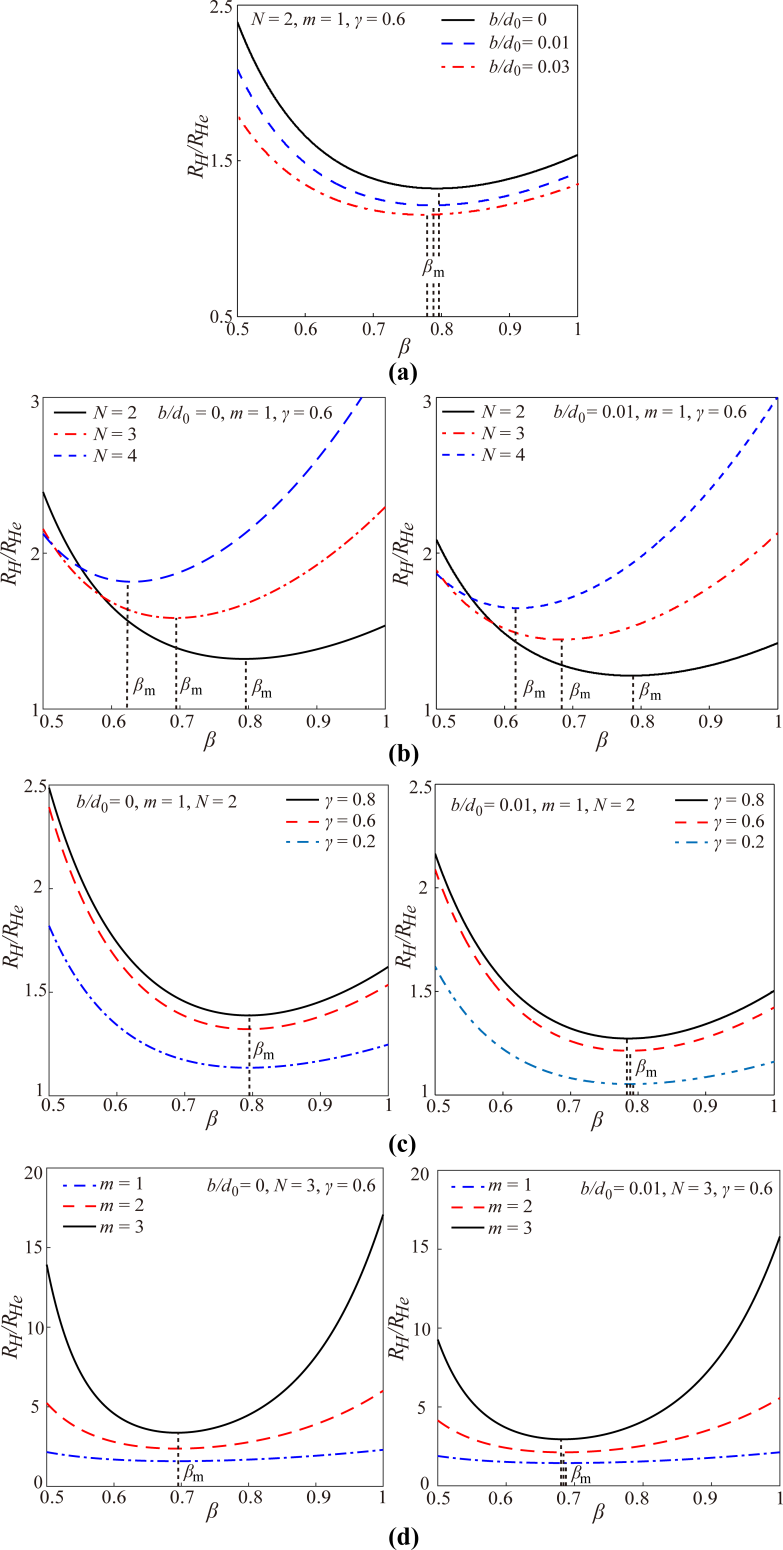
The dimensionless hydraulic resistance of laminar flow in a fractal tree-like microchannel network under both no-slip and slip conditions versus the diameter ratio β at different (a) ratios between slip length *b* and the diameter *d*_0_ of channel at the 0th level, (b) the branching number *N*, (c) the length ratio γ and (d) the level number *m.*

Based on Equation 11, Figure 3 gives the effect of the diameter ratio β between the daughter channel and the parent channel on the dimensionless hydraulic resistance of the laminar flow in the fractal tree-like microchannel network at different slip lengths *b*, branching numbers *N*, level numbers *m* and the length ratios γ between the daughter channel and the parent channel. In order to better illustrate the variation trend of the dimensionless hydraulic resistance, β is limited to a small range from 0.5 to 1. Figure 3 reveals that for both the no-slip (the case of *b*/*d*_0_ = 0 in Figure 3) and the slip condition (the cases of *b*/*d*_0_ ≠ 0 in Figure 3), the dimensionless hydraulic resistance first decreases and then increases with the increasing diameter ratio β. This means that there is an optimal diameter ratio β_m_, at which the hydraulic resistance reaches minimum when the diameter ratio between the daughter channel and parent channel is equal to β_m_. This optimal diameter ratio β_m_ is a significant parameter to guide the optimal design of fractal tree-like microchannel network for fluid flow to achieve the minimum hydraulic resistance. From Figure 3, it can be found that for the laminar flow in the fractal tree-like microchannel network under no-slip condition (the case of *b*/*d*_0_ = 0 in Figure 3), the optimal diameter ratio β_m_ is only dependent on the branching number *N* of the tree-like network. This is consistent with the well-accepted Murray’s law of β_m_ = *N*^−1/3^ and has been verified by both theoretical analyses and experimental studies [12,14–18]. However, Figure 3 also reveals that for the tree-like microchannel network with slip (the cases of *b*/*d*_0_ ≠ 0 in Figure 3), the optimal diameter ratio β_m_ to achieve the minimum hydraulic resistance is not only dependent on the branching number *N*, but also dependent on ratio between the slip length *b* and the diameter *d*_0_ of channel at the 0th level, the length ratio γ and the level number *m*. In other words, the existence of slip increases the complexity of the optimal design of a fractal tree-like microchannel network to achieve the minimum hydraulic resistance. Next, the effects of the slip length, the diameter of the channel at the 0th level, the branching number, the length ratio and the level number on the optimal diameter ratio will be analyzed in detail.

When comparing the dimensionless hydraulic resistance of the no-slip condition and the slip condition in Figure 3, it can be found that the slip can significantly reduce the dimensionless hydraulic resistance. From Figure 3a, when the diameter *d*_0_ of a channel at the 0th level remains constant, the increasing slip length can effectively reduce the dimensionless hydraulic resistance for the networks with the same structural parameters and dimensional parameters. This can be easily explained by Equation 4. Figure 3a also shows that the dimensionless hydraulic resistance stays constant with the varying diameter *d*_0_ of the channel at the 0th level for the no-slip condition. However, the dimensionless hydraulic resistance for the case of slip decreases with the decreasing diameter *d*_0_ of channel at the 0th level when the non-zero slip length stays constant. Additionally, it can be found that the effects of the branching number *N*, the length ratio γ and the level number *m* on the dimensionless hydraulic resistance for the case of slip is similar to the results for the case of no-slip [17].

**Figure 4:**
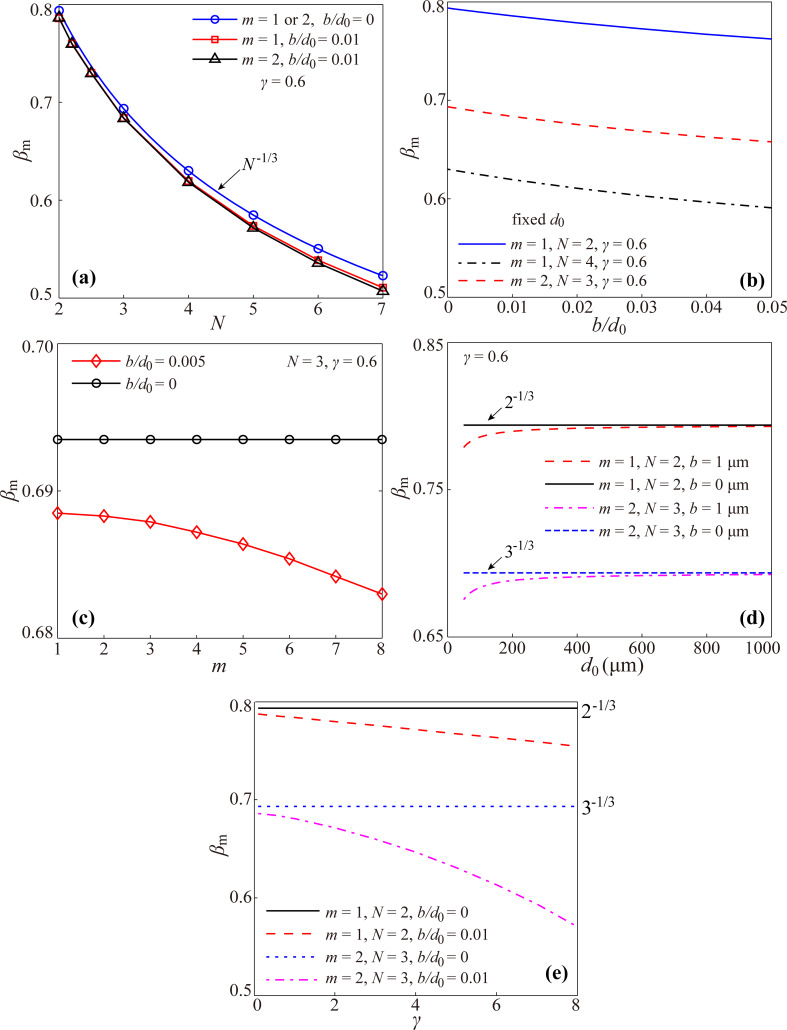
The optimal diameter ratio to achieve minimum hydraulic resistance versus (a) the branching number *N*, (b) slip length *b*, (c) the level number *m*, (d) the channel diameter *d*_0_ at the 0th level and (e) the length ratio γ.

Based on the above analysis, Figure 4 gives the effects of the slip length, the structural and dimensional parameters (the branching number, the level number, the length ratio and the diameter of channel at the 0th level) on the optimal diameter ratio to achieve the minimum hydraulic resistance for the laminar flow through a self-similar fractal tree-like microchannel network with slip conditions (the cases of *b*/*d*_0_ ≠ 0 in Figure 4). Furthermore, the effects of the branching number, the level number, the length ratio and the diameter of channel at the 0th level on the optimal diameter ratio for the laminar flow in the tree-like microchannel network with no-slip (the cases of *b*/*d*_0_ = 0 in Figure 4) are also given as comparison. All the curves in Figure 4 are obtained based on Equation 11.

All the results for the case of no-slip in Figure 4 reveal that the optimal diameter ratio for the laminar flow in the fractal tree-like microchannel network to achieve the minimum hydraulic resistance is independent of the level number, the length ratio and the diameter of channel at the 0th level and is only dependent on the branching number in a power function manner with a power of −1/3. This result obtained from Equation 11 is in good agreement with Murray’s law of β_m_ = *N*^−1/3^. This also verifies that the present model is correct.

However, for the case of slip, the results are much more complicated. From Figure 4a, the optimal diameter ratio still decreases with the increasing branching number *N* for slip conditions, but the relationship between the optimal diameter ratio and the branching number does not satisfy the strict manner of the power function with a power of −1/3. Figure 4a also indicates that both the slip length and the level number can affect the optimal diameter ratio for the tree-like network with slip to achieve minimum hydraulic resistance. In detail, Figure 4b shows that the optimal diameter ratio always decreases with increasing slip length for any tree-like network with different branching number and level number. Furthermore, Figure 4c shows that the optimal diameter ratio decreases with the increasing level number for the case of slip. Figure 4d shows that the optimal diameter ratio increases with increasing channel diameter at the 0th level for the fractal tree-like network with a fixed non-zero slip length, which seems completely different from the result for the no-slip condition. Actually, the results shown in Figure 4c and Figure 4d are consistent. Under a fixed non-zero slip length at every single channel wall, the diameter of the microchannels at the newly generated level decreases with the increase of level number, and the effect of slip on the fluid flow in the newly generated channels becomes stronger. Thus, a smaller optimal diameter ratio is needed for the microchannel with a larger level number to achieve the minimum hydraulic resistance. Figure 4e indicates that the optimal diameter ratio for the case of slip decreases with the increasing length ratio. This is also different from the result of the no-slip condition.

Additionally, both the results in Figure 4b and Figure 4d reflect that *b*/*d*_0_ is an important parameter that affects the optimal diameter ratio. With decreasing *b*/*d*_0_ or increasing *d*_0_/*b*, the optimal diameter ratio will gradually increase to approach the value of no-slip condition, that is, the complicated relations between the optimal diameter ratio and the structural and dimensional parameters simplify to that of Murray’s law, *N*^−1/3^. This is consistent with Equation 4, reflecting that the effect of slip on the fluid flow gradually diminishes with the increasing diameter of the channel. This is also the reason that the effect of slip on the fluid flow on the macroscale is usually neglected.

The present work indicates that although the slip can effectively reduce the hydraulic resistance as shown in Equation 4 and Figure 3, it increases the complexity of the optimal design of the fractal tree-like microchannel network to achieve the minimum hydraulic resistance for fluid flow. For the no-slip condition, there is a well-accepted optimization principle (i.e., Murray’s law with a simple expression of β_m_ = *N*^−1/3^) to guide the optimal design, and the unique factor that influences the optimal diameter ratio is the branching number. However, for the slip condition, every parameter of the slip length, the branching number, the level number, the length ratio and the diameter of channel at the 0th level can affect the optimal diameter ratio in a particular way, and there is no simple and clear mathematical relation between the optimal diameter ratio and the five influencing factors. However, using Equation 11, the optimal diameter ratio can be easily obtained. Additionally, it is noted that the complicated relations between the optimal diameter ratio and the structural and dimensional parameters for the slip condition become relaxed and simplify to that of Murray’s law when *b*/*d* is very small.

## Conclusion

In present paper, the effects of slip length *b*, as well as the structural and dimensional parameters (the branching number *N*, the level number *m*, the diameter ratio β of the daughter channel and the parent channel, the length ratio γ of the daughter channel and the parent channel and the diameter of channel at the 0th level *d*_0_) on the hydraulic resistance of laminar flow in a self-similar fractal tree-like microchannel network with slip condition are studied. It is found that there is an optimal diameter ratio β_m_ for the laminar flow in the fractal tree-like microchannel to achieve minimum hydraulic resistance. For the no-slip condition, the optimal diameter ratio is only dependent on the branching number in the manner β_m_ = *N*^−1/3^, namely, the well-known Murray’s law. However, for slip condition, this optimal diameter ratio is not only dependent on the branching number, but also dependent on the slip length, the level number, the length ratio and the diameter of channel at the 0th level. Furthermore, the optimal diameter ratio decreases with increasing slip length, increasing level number and increasing length ratio, but decreases with decreasing channel diameter at the 0th level. Thus, the optimal design of a fractal tree-like microchannel network to achieve the minimum hydraulic resistance for fluid flow should be carefully treated when slip condition is considered. Additionally, the complicated relations between the optimal diameter ratio and the structural and dimensional parameters become relaxed and simplify to Murray’s law when the ratio between slip length and diameter of channel is small enough.
